# Supplementary material for: TET activity safeguards pluripotency throughout embryonic dormancy
Source: Nat Struct Mol Biol. 2024 May 23;31(10):1625–39. doi: 10.1038/s41594-024-01313-7 (PMC11479945; doi:10.1038/s41594-024-01313-7)
Supplement: Supplementary file 1 — Supplementary table legends. [file 41594_2024_1313_MOESM1_ESM.pdf]

nature structural & molecular biology

Supplementary information https://doi.org/10.1038/s41594-024-01313-7

TET activity safeguards pluripotency throughout embryonic dormancy

In the format provided by the authors and unedited

This PDF file includes the legends for Supplementary Tables 1 and 2.

Supplementary Table 1
RNAseq normalized TPM values.

Supplementary Table 2
TET1 IP-MS results.
