# Supplementary material for: TET activity safeguards pluripotency throughout embryonic dormancy
Source: Nat Struct Mol Biol. 2024 May 23;31(10):1625–39. doi: 10.1038/s41594-024-01313-7 (PMC11479945; doi:10.1038/s41594-024-01313-7)
Supplement: Supplementary file 2 — Reporting Summary [file 41594_2024_1313_MOESM2_ESM.pdf]

nature portfolio

Corresponding author(s): Aydan Bulut-Karslioglu

Last updated by author(s): Mar 28, 2024

Reporting Summary

Nature Portfolio wishes to improve the reproducibility of the work that we publish. This form provides structure for consistency and transparency in reporting. For further information on Nature Portfolio policies, see our Editorial Policies and the Editorial Policy Checklist.

Statistics

For all statistical analyses, confirm that the following items are present in the figure legend, table legend, main text, or Methods section.

n/a	Confirmed	
☐	☒	The exact sample size (*n*) for each experimental group/condition, given as a discrete number and unit of measurement
☐	☒	A statement on whether measurements were taken from distinct samples or whether the same sample was measured repeatedly
☐	☒	The statistical test(s) used AND whether they are one- or two-sided<br>*Only common tests should be described solely by name; describe more complex techniques in the Methods section.*
☒	☐	A description of all covariates tested
☐	☒	A description of any assumptions or corrections, such as tests of normality and adjustment for multiple comparisons
☐	☒	A full description of the statistical parameters including central tendency (e.g. means) or other basic estimates (e.g. regression coefficient) AND variation (e.g. standard deviation) or associated estimates of uncertainty (e.g. confidence intervals)
☐	☒	For null hypothesis testing, the test statistic (e.g. *F*, *t*, *r*) with confidence intervals, effect sizes, degrees of freedom and *P* value noted<br>*Give P values as exact values whenever suitable.*
☒	☐	For Bayesian analysis, information on the choice of priors and Markov chain Monte Carlo settings
☒	☐	For hierarchical and complex designs, identification of the appropriate level for tests and full reporting of outcomes
☐	☒	Estimates of effect sizes (e.g. Cohen's *d*, Pearson's *r*), indicating how they were calculated

Our web collection on statistics for biologists contains articles on many of the points above.

Software and code

Policy information about availability of computer code

Data collection	Mass spectrometry: MaxQuant software (v1.6.10.43)<br>Imaging: Zen black and Zen blue software (version 2.3)<br>Flow cytometry: BD FACSDIVA Software v8.0.1 configuration 2B-5YG-3R-2UV-6V
Data analysis	Image analysis: CellProfiler v4.2.1<br>Plotting: GraphPad Prism v10, ggplot2 v3.3.5, survminer 0.4.9<br>R v4.1.0<br>TF footprinting: ATAC-seq peak regions were called by MACS2 (2.2.7.1) with 75-bp shift and 150-bp extension. Differential TF footprints inside these peak regions were identified by TOBIAS v0.12.11 using Homer motifs (v4.7, 8-25-2014).<br>MACS2 (2.1.2_dev)<br>BWA v0.7.17<br>cutadapt v2.4<br>GATK v4.1.4.1<br>STAR v2.7.5a<br>stringtie v2.0.6<br>BSMAP v2.90<br>samtools v1.10<br>MOABS v1.3.2<br>DEseq2 (version 1.38.2)<br>FlowJo (v10.8.2)

For manuscripts utilizing custom algorithms or software that are central to the research but not yet described in published literature, software must be made available to editors and reviewers. We strongly encourage code deposition in a community repository (e.g. GitHub). See the Nature Portfolio guidelines for submitting code & software for further information.

Data

Policy information about availability of data

All manuscripts must include a data availability statement. This statement should provide the following information, where applicable:

- Accession codes, unique identifiers, or web links for publicly available datasets
- A description of any restrictions on data availability
- For clinical datasets or third party data, please ensure that the statement adheres to our policy

All NGS data sets (WGBS, RNA-seq, ATAC-seq, CUT&TAG, FLASH) have been deposited on the GEO database: GSE221470 (https://www.ncbi.nlm.nih.gov/geo/query/acc.cgi?acc=GSE221470)
Proteomics data has been deposited to the ProteomeXchange Consortium (http://proteomecentral.proteomexchange.org) via the PRIDE partner repository with the dataset identifiers PXD039056.
Mouse reference genome (mm10) was used for mapping of sequencing data.

Field-specific reporting

Please select the one below that is the best fit for your research. If you are not sure, read the appropriate sections before making your selection.

☒ Life sciences ☐ Behavioural & social sciences ☐ Ecological, evolutionary & environmental sciences

For a reference copy of the document with all sections, see nature.com/documents/nr-reporting-summary-flat.pdf

Life sciences study design

All studies must disclose on these points even when the disclosure is negative.

Sample size	No sample size calculation was performed. All experiments were repeated at least twice. For quantifications of stainings, 150 to 2500 cells were used based on the availability of cell numbers in culture. No batch effects are observed and thus the data from biological replicates are combined. Use of embryos were minimized and a conventional sample size of 4-10 embryos are used for stainings. Survival analysis was performed on 50-100 embryos as performed previously in: van der Weijden, V.A., Stötzel, M., Iyer, D.P. et al. FOXO1-mediated lipid metabolism maintains mammalian embryos in dormancy. Nat Cell Biol 26, 181–193 (2024). https://doi.org/10.1038/s41556-023-01325-3
Data exclusions	No data were excluded.
Replication	All experiments are repeated at least twice independently and always with reproducible results.
Randomization	Samples were not randomized. For each biological replicate, corresponding time point samples are always processed and analyzed in parallel. Randomization was not possible due to different proliferation rates of cells.
Blinding	Investigators were not blinded to group allocation. The phenotype of the samples is obvious between proliferating and mTORi treated samples. Since all analysis is done via objective quantifications using software, blinding is not required.

Reporting for specific materials, systems and methods

We require information from authors about some types of materials, experimental systems and methods used in many studies. Here, indicate whether each material, system or method listed is relevant to your study. If you are not sure if a list item applies to your research, read the appropriate section before selecting a response.

Materials & experimental systems

n/a	Involved in the study	
☐	☒	Antibodies
☐	☒	Eukaryotic cell lines
☒	☐	Palaeontology and archaeology
☐	☒	Animals and other organisms
☒	☐	Human research participants
☒	☐	Clinical data
☒	☐	Dual use research of concern

Methods

n/a	Involved in the study	
☒	☐	ChIP-seq
☐	☒	Flow cytometry
☒	☐	MRI-based neuroimaging

Antibodies

Antibodies used	mESC and embryo stainings: Cells were then stained with primary antibodies 5hmC (ActifMotif, 39769; 1:200) and 5mC (Diagenode, C15200003; 1:100) overnight at 4 degree celsius.

The cells were washed thrice with wash buffer (PBS-T, 2% BSA) for 10 min.
donkey anti-rabbit AF647 (Thermo Fisher, A32795, 1:1000) and donkey anti-mouse AF488 (Thermo Fisher, A21202, 1:1000).
Western-blotting: Primaries: Flag antibody (Merck, F3165, 1:1000), TET1 (Novus Biologicals, NBP2-19290, 1:1000), TET2 (Cell Signaling Technology, 45010S, 1:1000). Secondaries: anti-rabbit (Thermo, 31460, 1:1000)
CUT&TAG: TET1 (Novus Biologicals, NBP2-19290, 1:100), TET2 (Cell Signaling Technology, 45010S, 1:50), TFE3 (Sigma HPA023881, 1:50), IgG (Abcam, ab46540, 1:100). Secondary: guinea pig α-rabbit antibody (ABIN101961, Antibodies online, 1:100)
Immunoprecipitation: Flag antibody (Merck, F3165, 8µg)
For flowcytometry Alexa488-SSEA1 antibody (Biolegend, 125610,1:1000) or Alexa647-AnnexinV antibody (Invitrogen, A23204,1:250) was used.

Validation

Validation statements by manufacturer:
5hmC (ActifMotif, 39769) Applications Validated by Active Motif: MeDIP: 0.1 - 0.5 µl per IP, DB: 1:10,000 dilution.
5mC (Diagenode, C15200003) Validated by DotBlot and IF.
Flag antibody (Merck, F3165, 1:1000) IP, IF, WesternBlot
TET1 (Novus Biologicals, NBP2-19290) Use in Flow Cytometry reported in scientific literature (PMID:34246869). IHC-P, ChIP assay-Assay dependent.
TET2 (Cell Signaling Technology, 45010S) WB-Western Blot, IP-Immunoprecipitation, IHC-Immunohistochemistry, ChIP-Chromatin Immunoprecipitation, C&R-CUT&RUN, C&T-CUT&Tag, DB-Dot Blot, eCLIP-eCLIP, IF-Immunofluorescence, F-Flow Cytometry
TFE3 (Sigma HPA023881), RNAi knockdown, IF
Alexa488-SSEA1 antibody (Biolegend, 125610) immunofluorescent staining with flow cytometric analysis.
Alexa647-AnnexinV antibody (Invitrogen, A23204) Cell Viability, Proliferation & FunctionCellular ImagingFlow Cytometry.
donkey anti-rabbit AF647 (Thermo Fisher, A32795) (Advanced Verification This Antibody was verified by Relative expression to ensure that the antibody binds to the antigen stated.)
donkey anti-mouse AF488 (Thermo Fisher, A21202)(Mouse IgG (H+L) Highly Cross-Adsorbed Secondary Antibody (A-21202) in ICC/IF)
As indicated by the Nature protocol article from the Henikoff laboratory (doi: 10.1038/s41596-020-0373-x, “Unlike ChIP-seq, in which antibodies bind their epitopes in solution, CUT&RUN and CUT&Tag bind chromatin targets in situ. Therefore, we expect that antibodies successfully tested for specificity by immunofluorescence (IF) are likely to work.”) Antibodies were validated via IF. Further every CUT&Tag experiments were performed with appropriate IgG controls to ensure specific cleavage.

Eukaryotic cell lines

Policy information about cell lines

Cell line source(s)

Mouse E14 cells: Sarah Kinkley Lab at the Max Planck Institute for Molecular Genetics
Wild-type KH2, Dnmt3a/b DKO: Alexander Meissner Lab
Tet1/2/3 TKO ESCs: Jacob Hanna Lab
HEK239T cells: Denes Hnisz Lab

Authentication

The cell lines were not authenticated.

Mycoplasma contamination

Cell lines tested negative for mycoplasma in regular tests.

Commonly misidentified lines
(See ICLAC register)

No commonly misidentified lines were used.

Animals and other organisms

Policy information about studies involving animals; ARRIVE guidelines recommended for reporting animal research

Laboratory animals

In vitro-fertilized (IVF) zygotes were electroporated to generate KOs, as previously described88. In brief, oocytes from superovulated B6D2F1 female mice (7-9 weeks old; Envigo) and sperm from F1B6xCAST were incubated for IVF, as previously described89. Pronuclei stage 3 (PN3) zygotes were rinsed with M2 (Sigma) and OptimMEM I (Gibco, 31985062) medium before electroporation 3 gRNAs per gene were designed targeting the first few exons. gRNAs were assembled with CAS9 into RNPs88. Embryos were electroporated on a NEPA21 (NEPAGENE, NEPA21) in a chamber with 5 mm electrode gap, and the following settings: 4 poring pulses with a voltage of 225 V, pulse length of 2 ms, pulse interval of 50 ms, decay rate of 10%, and uniform polarity, followed by 5 transfer pulses with a voltage of 20 V, pulse length of 50 ms, pulse interval of 50 ms, decay rate of 40%, and alternating polarity.
Electroporated zygotes were rinsed in KSOM drops (Merck, MR-106-D) and cultured until blastocyst stage. In the case of embryo transfer, 15 blastocysts were transferred into each uterine horn of pseudopregnant female CD-1 (21-25 g, Envigo, age 7-12 weeks mice 2.5 days post-coitum (dpc). E8.5 stage embryos were isolated from the uteri of foster mice. The embryos were dissected in 1X HBSS (Gibco) on ice after the decicuda were removed. Embryos were washed in 1X PBS (Gibco) with 0.4% BSA and imaged on an Axiozoom (ZEISS) microscope. Images were processed with Fiji.
For KO validation qPCRs, RNA was isolated using Arcturus Pico Pure RNA isolation kit (Biosystems) and reverse transcribed using High-Capacity cDNA synthesis kit (KAPA biosystems). RT-qPCR was done using Kapa SYBR 2x master mix. B-actin was used for normalization. qPCR results were visualized using GraphPad Prism v10.
In vitro diapause
ESCs or embryos were treated with the mTOR inhibitor INK128 or RapaLink-1 at 200 nM final concentration for the durations specified in individual experiments. To obtain embryos, 10- to 12-week-old b6d2F1 mice were superovulated via intraperitoneal injection with PMSG (5IU/100 µl) on day 0,with HCG 5 IU/100µl on day 2, and sacrificed on day 3. Oocytes were collected and incubated with 10 µl of motile sperm in CARD Medium (CosmoBio, KYD-003-EX) for in vitro fertilization. After overnight culture, 2-cell stage embryos were transferred to a fresh drop of KSOM (Merck, MR-107-D) and cultured until the blastocyst stage.
Animal experimentation
Animal experiments were performed according to local animal welfare laws and approved by authorities (Landesamt für Gesundheit

	und Soziales), covered by LaGeSo licenses ZH120, G0284/18, G021/19, and G0243/18-SGr1_G. Mice (7- to 12-week-old) were housed with enrichment material in ventilated cages (humidity 45-65%, temperature 20-24oC) on a 12h light/dark cycle and fed ad libitum.
Wild animals	No wild animals were used.
Field-collected samples	No samples were collected from the field.
Ethics oversight	All animal experiments were performed according to local animal welfare laws and approved by local authorities (Landesamt für Gesundheit und Soziales), covered by LaGeSo licenses ZH120, G0284/18, G021/19, and G0243/18-SGr1_G. Mice were housed in ventilated cages and fed ad libitum.

Note that full information on the approval of the study protocol must also be provided in the manuscript.

Flow Cytometry

Plots

Confirm that:

☒ The axis labels state the marker and fluorochrome used (e.g. CD4-FITC).

☒ The axis scales are clearly visible. Include numbers along axes only for bottom left plot of group (a 'group' is an analysis of identical markers).

☒ All plots are contour plots with outliers or pseudocolor plots.

☒ A numerical value for number of cells or percentage (with statistics) is provided.

Methodology

Sample preparation	Cells were dissocieted from plates, washed and resuspended in PBS containing 2% BSA and 5mM EDTA and kept on ice until sorting.
Instrument	FACS AriaFusion cell cytometer was used for analysis.
Software	Data were collected using BD FACSDIVA Software v8.0.1 and analyzed using FlowJo (v10.8.2).
Cell population abundance	Viable, single cells comprised ~90% of cells were sorted as input for RNAseq., ATAseq., and WGBS.
Gating strategy	FSC and SSC were used to set the first three gates to separate duplets from singlets by selecting the main population of cells and avoiding cell debris.

☒ Tick this box to confirm that a figure exemplifying the gating strategy is provided in the Supplementary Information.
