# Supplementary figures and images for: TET activity safeguards pluripotency throughout embryonic dormancy
Source: Nat Struct Mol Biol. 2024 May 23;31(10):1625–39. doi: 10.1038/s41594-024-01313-7 (PMC11479945; doi:10.1038/s41594-024-01313-7)

# Stoetzel et al, Extended Data Figure 3g source data

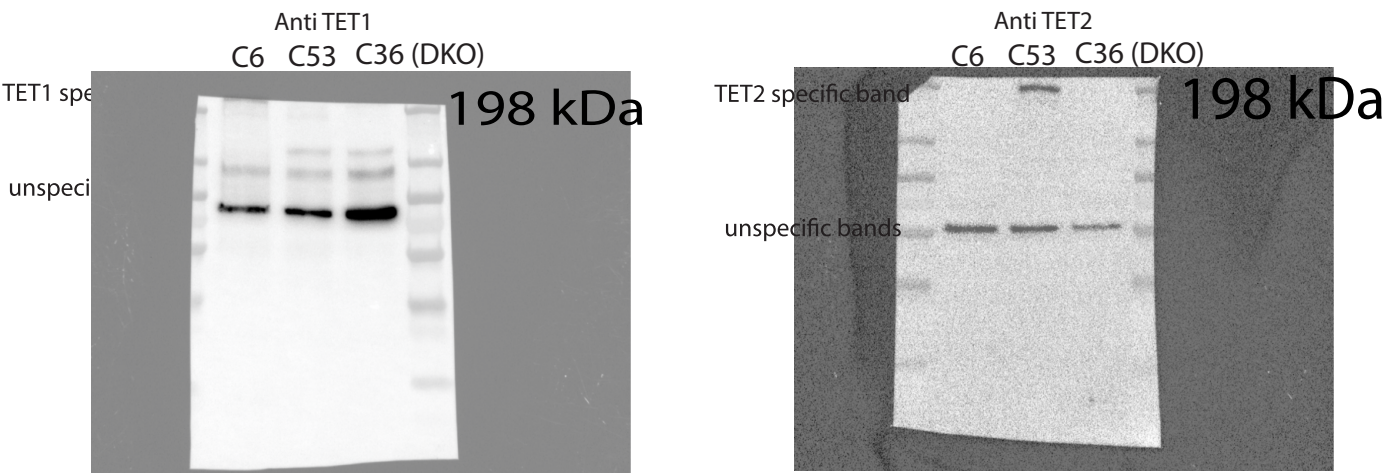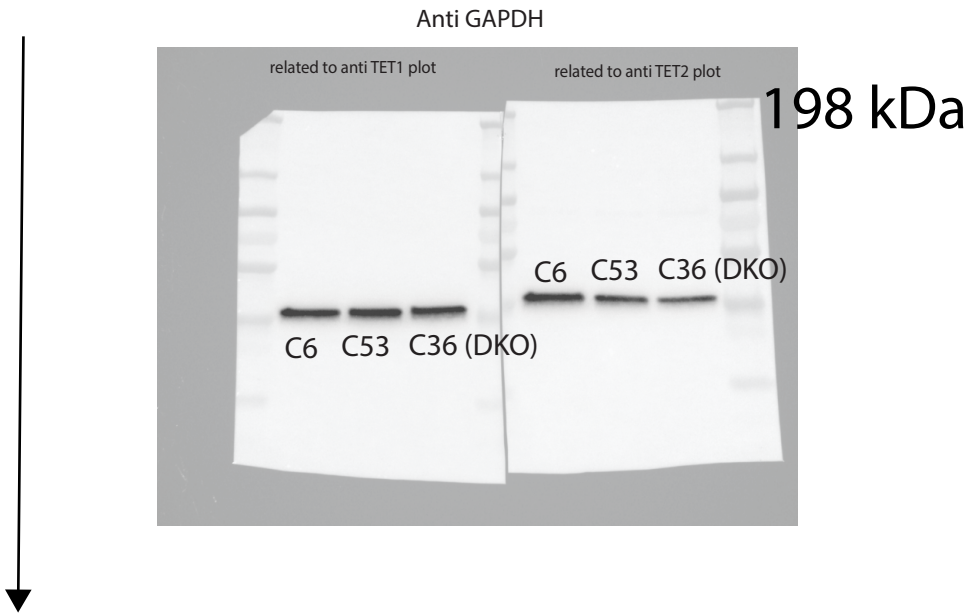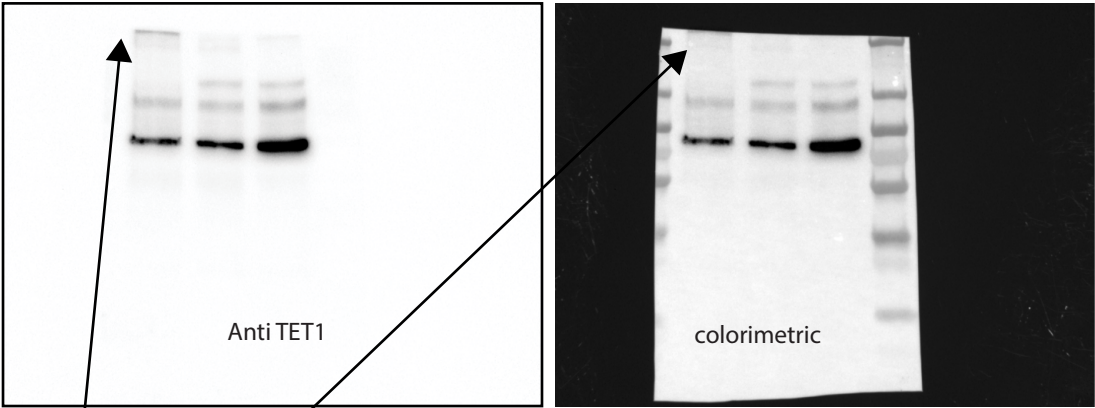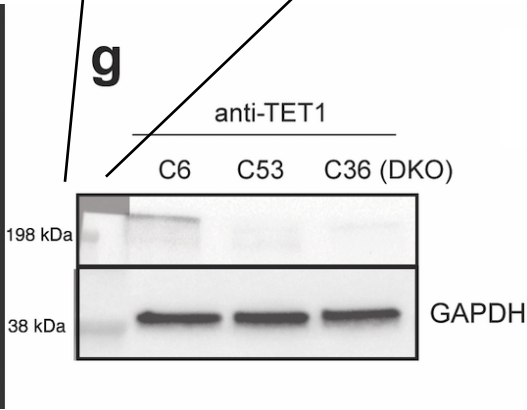

Supplement: Supplementary file 13 — Unprocessed western blots. [file 41594_2024_1313_MOESM13_ESM.pdf]

Stoetzel et al, Extended Data Figure 5e

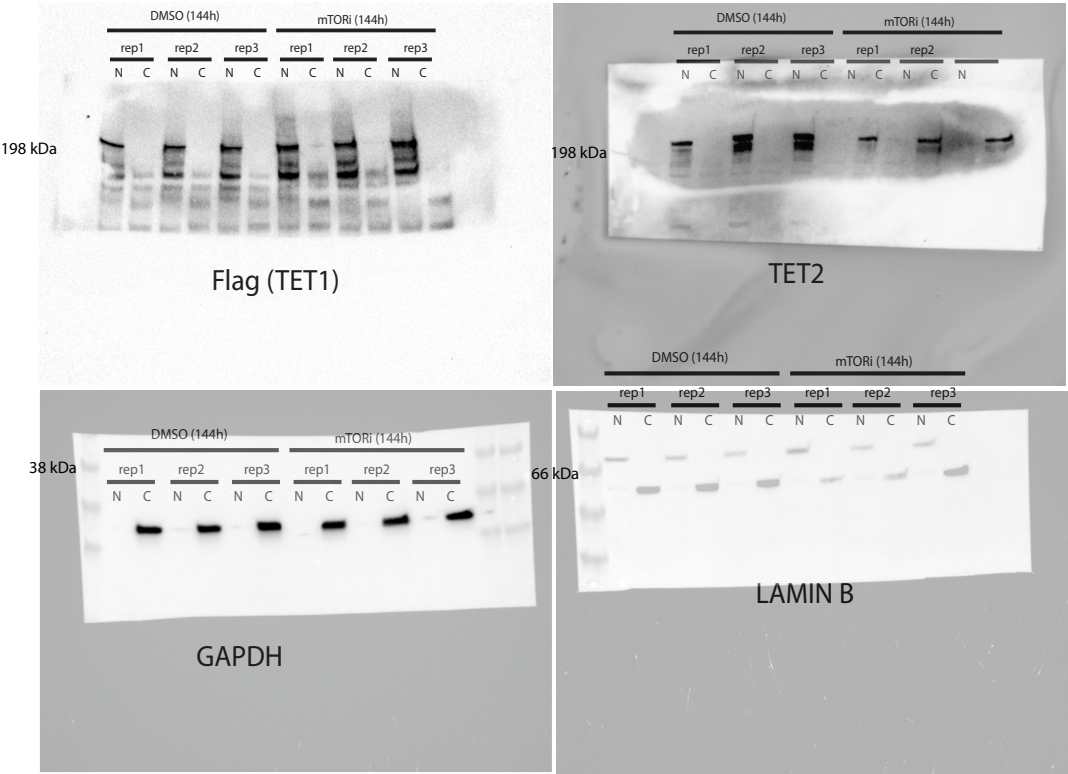

Supplement: Supplementary file 15 — Unprocessed western blots. [file 41594_2024_1313_MOESM15_ESM.pdf]

Stoetzel et al, Source Data Extended Data Figure 7b

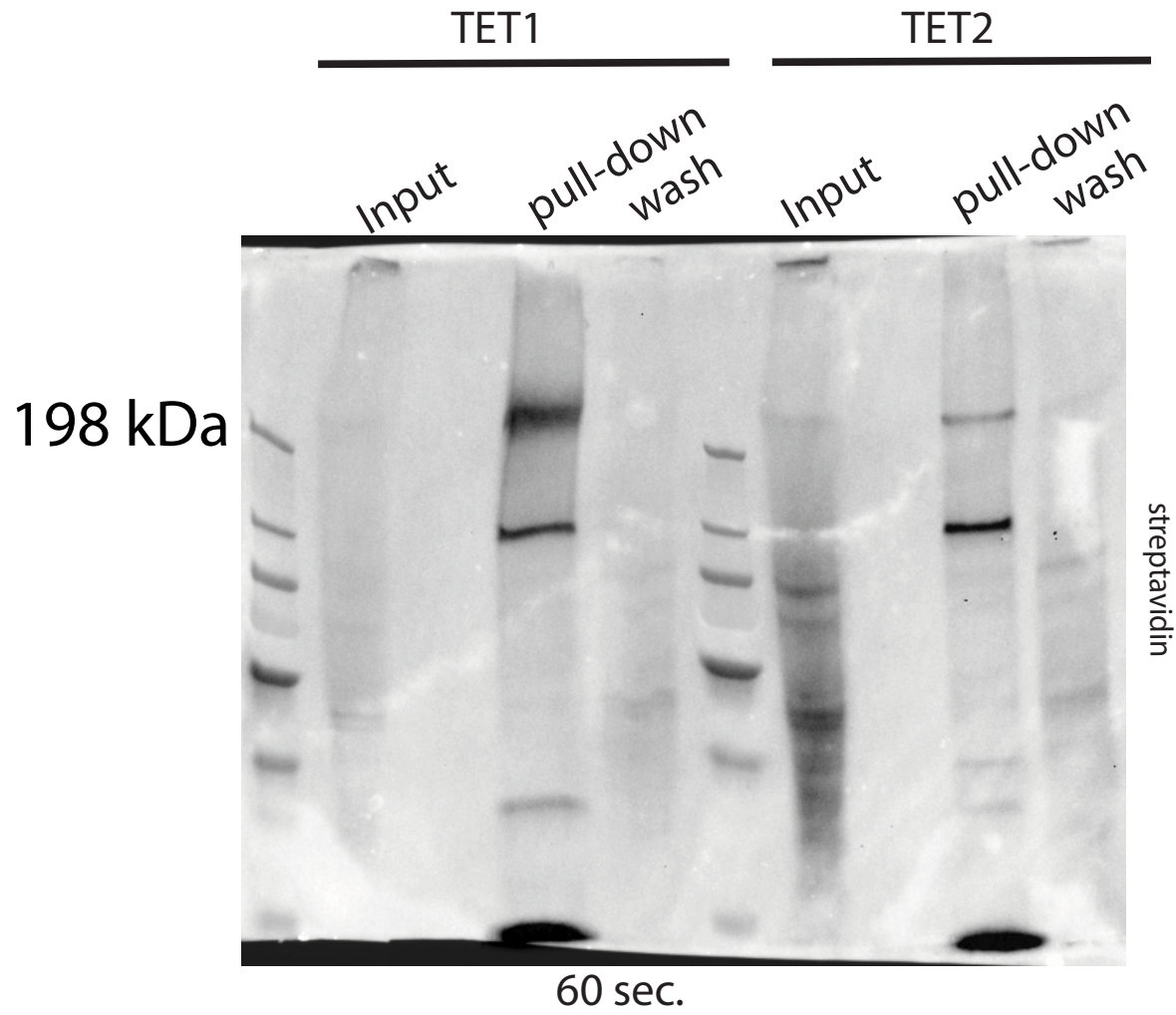

Supplement: Supplementary file 18 — Unprocessed western blots. [file 41594_2024_1313_MOESM18_ESM.pdf]
