# Supplementary material for: TET activity safeguards pluripotency throughout embryonic dormancy
Source: Nat Struct Mol Biol. 2024 May 23;31(10):1625–39. doi: 10.1038/s41594-024-01313-7 (PMC11479945; doi:10.1038/s41594-024-01313-7)

# Stoetzel et al, Source Data Extended Data Figure 6

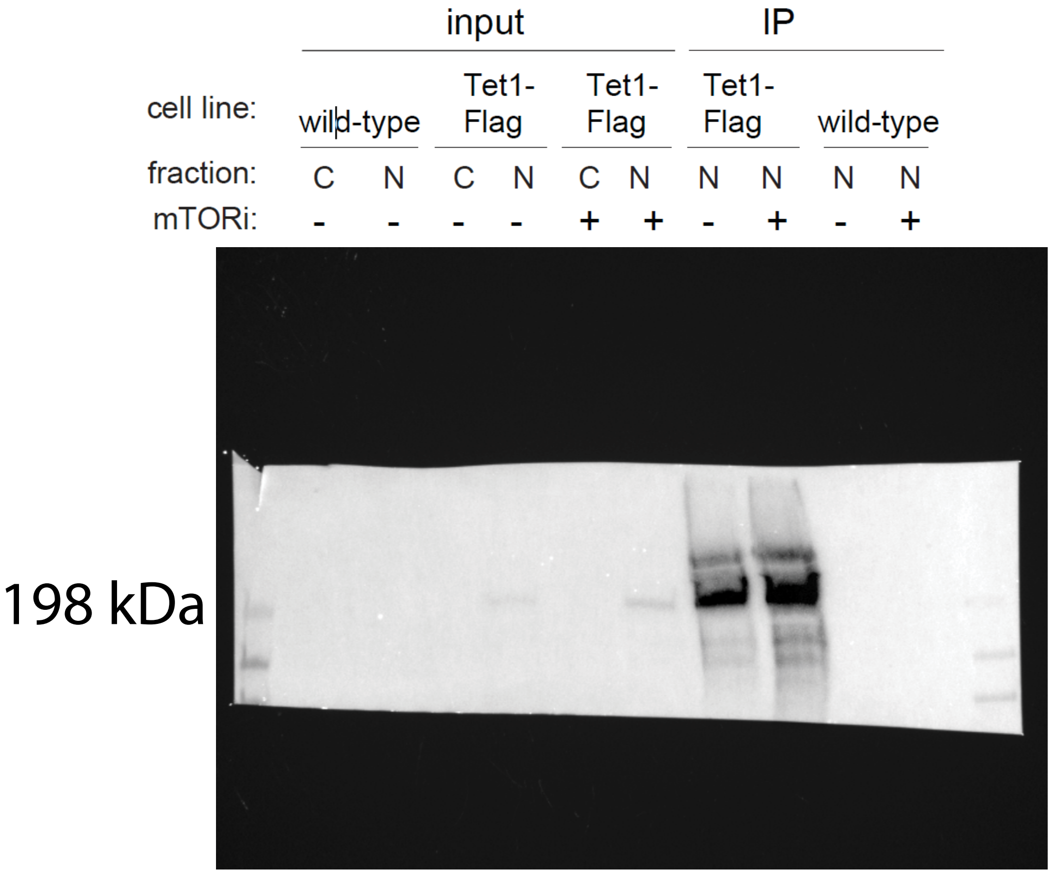

Supplement: Supplementary file 17 — Unprocessed western blots. [file 41594_2024_1313_MOESM17_ESM.pdf]
